# Changing Treatment May Affect the Predictive Ability of European Treatment Outcome Study Scoring for the Prognosis of Patients with Chronic Myeloid Leukemia

**DOI:** 10.4274/tjh.2016.0156

**Published:** 2017-03-01

**Authors:** Jing Huang, Leyan Wang, Lu Chen, He Qun, Xu Yajing, Chen Fangping, Zhao Xielan

**Affiliations:** 1 Xiangya Hospital, Central South University, Department of Hematology, Changsha, China

**Keywords:** Imatinib, Sokal score, Hasford score, European Treatment Outcome Study Score

## Abstract

**Objective:**

Previous studies compared the predictive ability of the European Treatment Outcome Study (EUTOS), Sokal, and Hasford scoring systems and demonstrated inconsistent findings with unknown reasons. This study was conducted to determine a useful scoring system to predict the prognosis of patients with chronic myeloid leukemia (CML) and identify the probable factors that affect the scoring.

**Materials and Methods:**

This is a retrospective cohort study. The predictive ability of EUTOS and the factors that affect scoring were analyzed in 234 Chinese chronic-phase CML patients treated with frontline imatinib, including a few patients temporarily administered hydroxyurea for cytoreduction before imatinib. Patients were stratified into different risk groups according to each scoring system to assess the treatment outcomes and the predictive ability of EUTOS scores between patients who received imatinib during the entire follow-up period and patients who received altered treatment because of intolerance, progression, and treatment failure.

**Results:**

Sixty-one (26.0%) patients received altered treatments during the follow-up. In the EUTOS low- and high-risk groups, the 5-year overall survival was 94.6% and 84.7% (p=0.011), 5-year event-free survival was 92.6% and 77.6% (p=0.001), and 5-year progression-free survival (PFS) was 95.3% and 82.4% (p=0.001), respectively. The predictive ability of EUTOS was better than that of the Sokal and Hasford scores (p=0.256, p=0.062, p=0.073) without statistical significance. All three scoring systems were valid in predicting early optimal response. Kaplan-Meier analysis showed a high association between overall PFS and the EUTOS scores in the standard-dose imatinib group (p<0.001).

**Conclusion:**

This study suggests that the EUTOS scoring system could predict the outcome of chronic-phase CML patients treated with standard-dose imatinib. Altered treatment is a crucial factor that affects the prognostic impact of EUTOS scoring. Achieving complete cytogenetic response at 18 months is an essential factor in predicting the prognosis of patients with CML.

## Abstract (tr)

**Amaç:**

Avrupa Tedavi İzlem Çalışması (EUTOS), Sokal ve Hasford skorlamalarının öngörüsel tahmin başarılarının karşılaştırıldığı çalışmalarda bilinmeyen nedenlerle tutarsız bulgular görülmektedir. Bu çalışma, kronik miyeloid lösemi (KML) hastalarının prognozunu tahmin edecek yararlı bir skorlama sistemi bulma ve skorlamayı etkileyen muhtemel faktörleri ortaya çıkartmak amacıyla yapılmıştır.

**Gereç ve Yöntemler:**

Bu çalışma geriye dönük hasta grubunda yapılmıştır. İmatinib tedavisi gören 234 Çinli kronik faz KML hastasında (birkaç hasta imatinib tedavisi öncesi kısa süreli hidroksiüre tedavisi almıştır) EUTOS tahmin skorları ve bu skorlamayı etkileyen faktörler incelenmiştir. Tedavi çıktılarını ve EUTOS skorlarının tahmin başarısını belirlemek amacıyla tüm izlem boyunca imatinib alan ve intolerans, ilerleme ya da tedavi başarısızlığı gibi nedenlerle farklı tedaviler alan hastalar, her skorlama sistemine göre değişik risk gruplarında sınıflandırılmıştır.

**Bulgular:**

Takip sürecinde hastaların 61 tanesinde (%26) tedavi değişikliği olmuştur. EUTOS düşük ve yüksek risk gruplarında 5 yıllık genel sağkalım sırasıyla %94,6 ve %84,7 (p=0,011), hastalıksız sağkalım %95,3 ve %82,4 (p=0,001) ve progresyonsuz sağkalım %95,3 ve %77,6 (p=0,001) olarak bulunmuştur. EUTOS tahminleri, istatistiksel olarak anlamlı olmamakla beraber, Sokal ve Hasford skorlarından daha iyi görülmektedir (p=0,256, p=0,062, p=0,073). Her üç skorlama da erken optimal yanıtın değerlendirmesinde geçerli görülmektedir. Kaplan-Meier analizleri, standart doz imatinib kullanılan gruplarda, genel progresyonsuz sağkalım ve EUTOS skorları arasında yüksek anlamlılıkta bir ilişki göstermektedir (p<0,001).

**Sonuç:**

Bu çalışma EUTOS skorlama tahmin sisteminin, standart-doz imatinib ile tedavi edilen kronik faz KML hastalarının yanıtlarının değerlendirmesinde uygun olduğunu göstermektedir. Tedavi değişikliği, EUTOS skorlamasının prognostik değerlendirme sonucunu etkileyen önemli bir faktördür. KML hastalarının prognoz tahminleri etkileyen bir diğer önemli faktör de 18. ayda tam sitogenetik yanıta ulaşılmasıdır.

## INTRODUCTION

As the firstline treatment for chronic myeloid leukemia (CML), imatinib is widely used after diagnosis and dramatically improves the overall survival (OS) of CML patients [1]. Predicting the prognosis is significant for the management of CML patients. Currently, the European Treatment Outcome Study (EUTOS), Hasford, and Sokal prognostic scoring systems are used for predicting the prognosis of CML patients [2,3,4]. The EUTOS scoring system is a novel prognostic scoring system that challenges the conventional Sokal and Hasford scoring systems in predicting the outcome of CML patients. However, recent studies examining the effectiveness of the EUTOS scoring system in predicting the prognosis of CML patients showed controversial results. For example, several studies from different regions of the world compared the clinical significance of the three prognostic scoring systems. Five studies found that EUTOS was better than the Hasford and Sokal systems in predicting the prognosis of CML patients [1,2,3,5,6]. In contrast, 3 studies showed that the EUTOS score does not predict prognosis in CML patients [7,8,9]. It is currently unknown what factors caused these controversial findings.

The purpose of this study was to compare the predictive ability of the Sokal, Hasford, and EUTOS prognostic scoring systems by stratifying CML-chronic-phase (CP) patients who received firstline imatinib mesylate at diagnosis into different risk groups. The possible factors that affect the prognostic ability of EUTOS were further explored according to the three scoring systems.

## MATERIALS AND METHODS

### Patients

A total of 234 CML-CP patients (162 males, 72 females) who received imatinib mesylate (Novartis Oncology, Novartis Pharma Stein AG, Stein, Switzerland) treatment within 6 months of diagnosis at X Hospital between January 2004 and July 2014 were recruited for this study. CML-CP was diagnosed according to published diagnostic criteria [10], and all patients were treated with a standard dose of imatinib (400 mg/day) over 3 months. No other treatment was given, except for hydroxyurea temporarily administered for cytoreduction before imatinib in 9 patients.

### Calculations of the Chronic Myeloid Leukemia Prognostic Indexes

The Sokal score was calculated using the following formula: Exp 0.0116 × (age in years-43.4) + 0.0345 × (spleen size-7.51) + 0.188 × [(platelet count/700)^2^-0.563] + 0.0887 × (blast cells-2.10). Patients with a score of less than 0.8 were assigned to the low Sokal risk group, patients with a score from 0.8 to 1.2 were assigned to the intermediate Sokal risk group, and patients with a score greater than 1.2 were assigned to the high Sokal risk group [11]. The Hasford score was calculated as follows: 0.666 (when age >50 years) + (0.042×spleen size) + 1.0956 (when platelet count >1500×10^9^/L) + (0.0584×blast cell count) + 0.20399 (when basophil count >3%) + (0.0413×eosinophil count) × 100. Patients with a score of less than 780 were assigned to the low Hasford risk group, patients with a score from 781 to 1480 were assigned to the intermediate Hasford risk group, and patients with a score higher than 1480 were assigned to the high Hasford risk group [12]. The EUTOS score was calculated as follows: (7×basophil count) + (4×spleen size), where the spleen was measured in centimeters below the costal margin and basophils as a percentage rate. Patients with a EUTOS score higher than 87 were assigned to the high EUTOS risk group, while patients with a EUTOS score of less than or equal to 87 were assigned to the low EUTOS risk group [10].

### Definitions

OS: the length of time from the date of diagnosis to the date of death or final follow-up (1 July 2014).

Event-free survival (EFS): the length of time from the date of initiating imatinib therapy to the date of failure according to the European Leukemia Net criteria, the date of stopping treatment due to imatinib intolerance, or the date of last follow-up in patients whose treatments did not fail [13].

Progression-free survival (PFS): the length of time from the date of imatinib therapy initiation to the date of progression to accelerated phase (AP)/blastic phase (BP) or to the date of death.

Complete cytogenetic response (CCyR): no Philadelphia chromosome was detected in the patient by G-banding analysis of bone marrow and no Philadelphia cell was detected in the patient when using fluorescence in situ hybridization analysis of peripheral blood.

Partial cytogenetic response (PCyR): 1%-35% Philadelphia chromosome in a patient’s bone marrow.

Major molecular response (MMR): the achievement of ≥3 logs reduction in BCR-ABL mRNA from the standardized baseline [14,15,16,17].

### Statistical Analysis

Data were analyzed using SPSS 17.0 (SPSS Inc., Chicago, IL, USA). Normally distributed continuous variables were presented as mean ± standard deviation, and non-normally distributed continuous variables were presented as medians with interquartile ranges. Kaplan-Meier methods and log rank tests were applied to analyze the time-to-event data. The 5-year EFS, PFS, and OS and the cumulative incidence of PCyR, CCyR, and MMR were compared using the chi-square test. A value of p<0.05 was considered statistically significant.

## RESULTS

### Baseline Characteristics

The mean age at diagnosis was 40.3±13.2 years. Median follow-up duration of imatinib treatment was 20.5 months (range: 9-120 months) months. The median duration from diagnosis of CML to imatinib initiation was 19 (range: 6-115) days. The CCyR rates at 12 months and 18 months were 56.4% and 65.4%, respectively.

According to the EUTOS scoring system, 149 patients (63.7%) were classified as low-risk and 85 patients (36.3%) were classified as high-risk. Using the Sokal scoring system, 66, 70, and 98 patients were classified into low-, intermediate-, and high-risk groups, while 94, 93, and 47 patients were classified into low-, intermediate-, and high-risk groups when the Hasford scoring system was used. The baseline characteristics of patients with CML-CP are shown in Table 1.

### Outcomes

**Figure 1:**
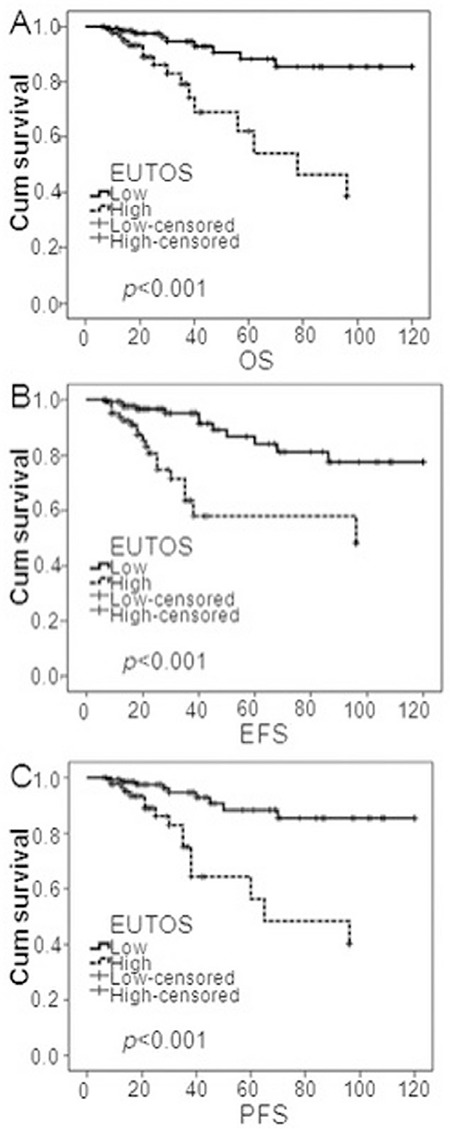
Overall survival, event-free survival, and progression-free survival using the European Treatment Outcome Study score system. There was a significant difference in overall survival (A), event-free survival (B), and progression-free survival (C) between the risk groups (p<0.001, p<0.001, p<0.001). EUTOS: European Treatment Outcome Study, OS: overall survival, EFS: event-free survival, PFS: progression-free survival.

The 5-year OS, EFS, and PFS of the 234 patients were 91.0%, 87.2%, and 90.6%, respectively. The 5-year OS, EFS, and PFS were 94.6% and 84.7% (p=0.011), 92.6% and 77.6% (p=0.001), and 95.3% and 82.4% (p=0.001) for low and high EUTOS risk groups, respectively. Significant differences in 5-year OS, EFS, and PFS were observed between the low EUTOS and high EUTOS risk groups. However, there were no significant differences between groups classified by the Sokal (p=0.137, p=0.106, p=0.110, respectively) and Hasford (p=0.256, p=0.062, p=0.073, respectively) prognostic scoring systems. Moreover, Kaplan-Meier analysis of OS, EFS, and PFS showed significant differences between the low and high EUTOS risk groups (p<0.001, p<0.001, p<0.001) (Figure 1), but no significant difference was found between Sokal groups (p=0.335, p=0.123, p=0.170 for low, intermediate-, and high-risk groups) or Hasford groups (p=0.135, p=0.057, p=0.052 for low-, intermediate-, and high-risk groups).

The overall rates of PCyR at 3 months, CCyR at 12 months and 18 months, and MMR at 18 months for all CML patients were 18.4%, 56.4%, 65.4%, and 46.2%, respectively. Furthermore, 131 patients (87.9%) and 22 patients (25.9%) achieved CCyR at 18 months in the low and high EUTOS risk groups (p<0.001); 60 patients (90.9%), 59 patients (84.3%), and 34 patients (34.7%) achieved CCyR at 18 months in the low, intermediate, and high Sokal risk groups (p<0.001), respectively; and 84 (89.4%), 57 (61.3%), and 12 patients (25.5%) achieved CCyR at 18 months in the low, intermediate, and high Hasford risk groups (p<0.001), respectively. As shown in Table 2, PCyR was significantly validated at 3 months in all 3 prognostic scoring systems (p<0.001), CCyR was significantly validated at 12 months and 18 months (p<0.001), and MMR was significantly validated at 18 months in all 3 prognostic scoring systems (p<0.001).

Overall, of the 234 CML-CP patients, 173 patients (73.9%) were treated with the standard dose (400 mg) of imatinib, while 61 patients (26.0%) received altered treatments because of intolerance, progression, or treatment failure. The rates of 5-year PFS were 97.5% and 87.3% (p=0.012) for patients treated with standard-dose imatinib in the EUTOS low-risk (118, 68.2%) and high-risk (55, 31.7%) groups, but only 87.1% and 73.3% (p=0.176) for patients who received altered treatments in low (31, 50.8%) and high EUTOS risk groups (30, 49.1%), respectively.

**Figure 2:**
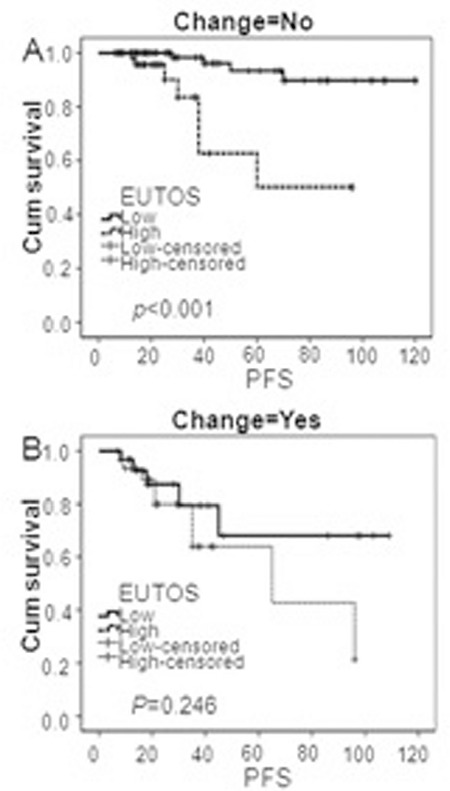
Progression-free survival using European Treatment Outcome Study score for chronic myeloid leukemia-chronic-phase patients who received imatinib or altered treatment. (A) Progression-free survival using European Treatment Outcome Study score for chronic myeloid leukemia-chronic-phase patients treated with standard-dose imatinib. There was a significant difference between the risk groups (p<0.001). (B) Progression-free survival using European Treatment Outcome Study score for chronic myeloid leukemia-chronic-phase patients who received altered treatment. There was no significant difference between the risk groups (p=0.246).

There were significant differences in 5-year OS between low and high EUTOS risk score groups in patients that received standard-dose imatinib treatment (97.5% and 89.1%, p=0.03), whereas it was not different in the patients who received altered treatments (83.9% and 76.7%, p=0.479). Furthermore, Kaplan-Meier analysis showed a high association between overall PFS and EUTOS scores in patients who received standard-dose imatinib treatment (p<0.001), but no significant correlation in patients who received altered treatment (p=0.246) (Figure 2).

## DISCUSSION

Previous studies presented controversial findings on whether EUTOS is more useful in predicting the survival of CML patients than the Sokal and Hasford scoring systems. This study demonstrated that the EUTOS, Hasford, and Sokal scoring systems were all effective in predicting early optimal response (PCyR at 3 months, CCyR at 12 months and 18 months, and MMR at 18 months) in CML-CP patients who were treated with imatinib as frontline therapy, but the EUTOS scoring system was more effective as a prognostic indicator of OS, EFS, and PFS than the Sokal and Hasford scoring systems. A novel finding in this study is that altered treatment is a key factor that affects the prognostic ability of the EUTOS scoring system.

Among the 3 studies not supporting the predictive effect of EUTOS in the prognosis of CML patients [7,8,9], 33.6% of 282 CML-CP patients in the study of Marin et al. were treated with imatinib as frontline therapy, but the treatment was changed to a second-generation tyrosine kinase inhibitor (2^nd^ TKI) [9]. In the study of Jabbour et al. [8], 84.7% of 465 CML-CP patients were treated with high-dose imatinib (44.7%) and a 2^nd^ TKI (40%) at diagnosis. In the study of Yamamoto et al., poor patient adherence to imatinib therapy was mentioned [7]. Among the studies supporting a positive effect of EUTOS in predicting the prognosis of CML patients, only 21.0% of 1288 CML patients in the study of Hoffmann et al. [18] and 25.0% of 2060 patients in the study of Hasford et al. [6] received high-dose imatinib (600-800 mg), while no patients received 2^nd^ TKI therapy. In this study, 26.0% patients received altered treatment during follow-up (20.5% received low-dose imatinib; 5.5% were switched to a 2^nd^ TKI). The percentage of patients receiving altered treatments in our study is comparable to those of the studies supporting a positive effect of EUTOS, but lower than that of studies supporting a negative effect. In this study, we found that 5-year PFS and OS had no significant correlations with EUTOS scores in patients who received altered treatments, but they were significantly associated with EUTOS scores in patients without altered treatments. Thus, changing treatment may be a key factor that affects the predictive ability of EUTOS. In addition, the percentage of patients in the high EUTOS risk group was small in the three negative studies (11.2% in the Marin et al. [9] study, 8% in the Jabbour et al. [8] study, and 11% in the Yamamoto et al. [7] study), while it was high (36.3%) in the present study. We propose that the small number of patients in the high-risk group in the three negative studies may have caused a bias.

The end-points for assessment of the EUTOS scoring system may also influence the conclusions. The majority of previous studies analyzed the overall CCyR, but only 3 reports [6,7,18] assessed CCyR at 18 months. In this study, the OS, EFS, and PFS were evaluated as long-term response, while PCyR at 3 months, CCyR at 12 months and 18 months, and MMR at 18 months were evaluated as early optimal response according to the 2013 National Comprehensive Cancer Network guidelines. This study demonstrated that the rates of 5-year PFS in patients achieving CCyR at 18 months was significantly higher than that in patients not achieving CCyR at 18 months (99.3% vs. 74.1%, p<0.001). Therefore, CML patients who did not achieve CCyR at 18 months were more likely to progress AP/BP or death and CCyR at 18 months should be considered as an essential end-point to assess the survival of CML patients.

## CONCLUSION

In conclusion, the EUTOS prognostic scoring system is an effective prognostic tool in assessing the outcome of CML-CP patients who were treated with standard-dose imatinib. Altered treatment should be considered as a key factor when using the EUTOS scoring system. CCyR at 18 months during therapy is an essential end-point to assess the survival of CML-CP patients. Our findings may have important clinical implications, but they should be confirmed in a larger cohort and validated in a prospective observational study.

## Figures and Tables

**Table 1:**
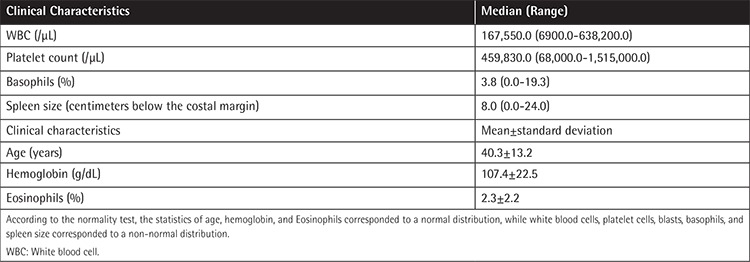
Clinical characteristics of patients at diagnosis (n=234).

**Table 2:**
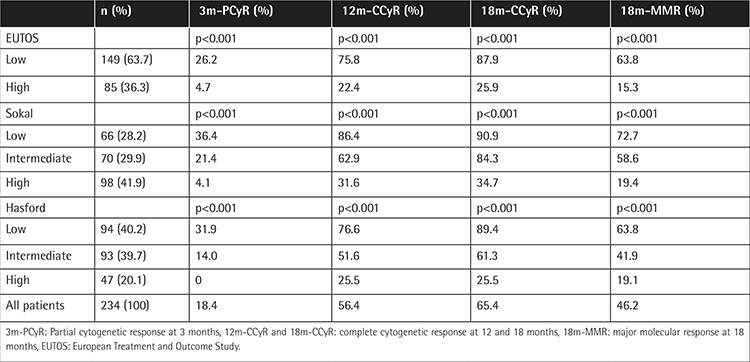
Comparison of Sokal, Hasford, and European Treatment Outcome Study scores for curative effects.
